# Vulnerability to chronic stress and the phenotypic heterogeneity of presbycusis with subjective tinnitus

**DOI:** 10.3389/fnins.2022.1046095

**Published:** 2022-12-21

**Authors:** Jian Ruan, Xiuhua Hu, Yuehong Liu, Zhao Han, Qingwei Ruan

**Affiliations:** ^1^Department of Otolaryngology, Huadong Hospital, Shanghai Medical College, Fudan University, Shanghai, China; ^2^Laboratory of Aging, Anti-aging & Cognitive Performance, Shanghai Institute of Geriatrics and Gerontology, Huadong Hospital, Fudan University, Shanghai, China; ^3^Shanghai Key Laboratory of Clinical Geriatrics, Research Center of Aging and Medicine, Huadong Hospital, Shanghai Medical College, Fudan University, Shanghai, China

**Keywords:** presbycusis, tinnitus, heterogeneity, chronic stress, allostatic load, frailty phenotype, comorbidity

## Abstract

Age-related functional reserve decline and vulnerability of multiple physiological systems and organs, as well as at the cellular and molecular levels, result in different frailty phenotypes, such as physical, cognitive, and psychosocial frailty, and multiple comorbidities, including age-related hearing loss (ARHL) and/or tinnitus due to the decline in auditory reserve. However, the contributions of chronic non-audiogenic cumulative exposure, and chronic audiogenic stress to phenotypic heterogeneity of presbycusis and/or tinnitus remain elusive. Because of the cumulative environmental stressors throughout life, allostasis systems, the hypothalamus-pituitary-adrenal (HPA) and the sympathetic adrenal–medullary (SAM) axes become dysregulated and less able to maintain homeostasis, which leads to allostatic load and maladaptation. Brain–body communication via the neuroendocrine system promotes systemic chronic inflammation, overmobilization of energetic substances (glucose and lipids), and neuroplastic changes via the non-genomic and genomic actions of glucocorticoids, catecholamines, and their receptors. These systemic maladaptive alterations might lead to different frailty phenotypes and physical, cognitive, and psychological comorbidities, which, in turn, cause and exacerbate ARHL and/or tinnitus with phenotypic heterogeneity. Chronic audiogenic stressors, including aging accompanying ontological diseases, cumulative noise exposure, and ototoxic drugs as well as tinnitus, activate the HPA axis and SAM directly and indirectly by the amygdala, promoting allostatic load and maladaptive neuroplasticity in the auditory system and other vulnerable brain regions, such as the hippocampus, amygdala, and medial prefrontal cortex (mPFC). In the auditory system, peripheral deafferentation, central disinhibition, and tonotopic map reorganization may trigger tinnitus. Cross-modal maladaptive neuroplasticity between the auditory and other sensory systems is involved in tinnitus modulation. Persistent dendritic growth and formation, reduction in GABAergic inhibitory synaptic inputs induced by chronic audiogenic stresses in the amygdala, and increased dendritic atrophy in the hippocampus and mPFC, might involve the enhancement of attentional processing and long-term memory storage of chronic subjective tinnitus, accompanied by cognitive impairments and emotional comorbidities. Therefore, presbycusis and tinnitus are multisystem disorders with phenotypic heterogeneity. Stressors play a critical role in the phenotypic heterogeneity of presbycusis. Differential diagnosis based on biomarkers of metabonomics study, and interventions tailored to different ARHL phenotypes and/or tinnitus will contribute to healthy aging and improvement in the quality of life.

## 1 Introduction

Presbycusis, also referred to as ARHL, is the most common sensory impairment with phenotypic heterogeneity in older adults. The etiology, pathophysiology, and clinical characteristics of ARHL and tinnitus are heterogeneous. Some individuals might only have presbycusis with different levels of deficits in peripheral and/or CAP, which affects normal speech comprehension in quiet and/or noisy environments. Other individuals may have other concurrent hearing problems, including hyperacusis, tinnitus, and auditory hallucinations. More individuals concurrently present with other physical and psychosocial comorbidities, including cardiovascular disease, diabetes, cognitive impairment, AD, depression, anxiety, psychiatric conditions, and different frailty phenotypes including physical, cognitive, and psychosocial frailty. Although the causality between ARHL and/or tinnitus and the above-mentioned comorbidities and frailty phenotypes has been poorly established, accumulative epidemiological studies suggest that ARHL and/or tinnitus are closely related to these comorbidities and frailty phenotypes (Panza et al., 2015; Wayne and Johnsrude, 2015; Yoo et al., 2019; Ruan et al., 2021). Moreover, some comorbidities, such as cardiometabolic multimorbidity (Tai et al., 2022), and psychological disorders (Price and Duman, 2020) have been independently associated with the risk of dementia.

**FIGURE 1:**
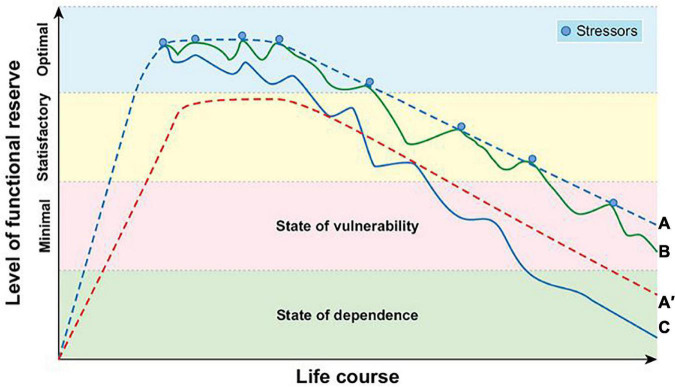
Schematic representation of life-course trajectories of functional reserve and vulnerability to stressors. **(A)** Normal development and decline of functional reserve. **(A′)** Reduced functional reserve due to suboptimal development at early life. **(B)** Trajectories of adaptive response to stressors. **(C)** Trajectories of maladaptive response to stressors.

The phenotypic heterogeneity of presbycusis and/or tinnitus depends on the functional reserve and vulnerability to stressors in different organs/systems. The functional reserve of an organ or system refers to its ability to successfully return to its original physiological state following repeated episodes of stress. Functional reserve comprise multiple domains, including physiological (different organs), cognitive, psychosocial, and relational reserves, as well as auditory reserves. Functional reserve displays rapid growth and development during the growth phase, including conception, prenatal, prepubertal, and pubertal periods, reaching a plateau at the maturity phase, and then gradually declining with age at the senescence phase (Kuh et al., 2014; Ben-Shlomo et al., 2016) (Figure 1). Functional reserve heterogeneity depends on intrinsic (passive) and extrinsic (active) constructs and plasticity (Ben-Shlomo et al., 2016; Cullati et al., 2018). The intrinsic constructs, the hardware of the organ/system, are genetically determined, which mainly influences the initial level of life course trajectories of function. At the molecular level, several metabolic pathways, such as the bioenergetic pathways and antioxidant systems, exhibit excess metabolic capacity (Atamna et al., 2018). The extrinsic constructs and the organ/system software, which are determined by life experiences, also modify the life course trajectories of function and vulnerability to stressors. Intellectual stimulation through engaging in educational, occupational, or leisure activities can increase the cognitive reserve and delay cognitive aging as well as improve cognitive function, age-related changes in brain structure, and reduce the risk of developing dementia (Valenzuela and Sachdev, 2006; Zahodne et al., 2015; Stern et al., 2019). Music training and being bilingual can improve cognitive and auditory reserves such as those related to brainstem encoding and sensory acuity, and decrease the auditory function decline due to sensorineural deafness and aging or diseases (Skoe and Kraus, 2014; Kroll et al., 2015; Krizman et al., 2016). Individuals with greater functional reserve can maintain higher functioning of the organ/system, even if the pathology is advancing. During critical periods, such as gestation, childhood, and adolescence, the effect of experiences on functional reserve trajectories is particularly powerful (Kuh et al., 2014; Skoe and Kraus, 2014; Jafari et al., 2020b; McEwen and Akil, 2020). The influence of experience on sensory, cognitive, and psychosocial processing declines gradually with increasing age.

Vulnerability is defined as a lack of functional reserve (below a clinical threshold) and reduced capacity of an individual to maintain or regain functional reserve during or after adverse events or stressors (Cullati et al., 2018) (Figure 1). An important characteristic of aging is the decline in functional reserve and the increase in vulnerability to stressors. A tolerable small insult, such as a minor infection or surgery, can result in a dramatic and disproportionate change in the health state of some aging individuals (Clegg et al., 2013). These individuals cannot maintain or regain functional reserve. At the individual level, when the decline of functional reserve in multiple physiological systems is accompanied by an increased vulnerability to homeostatic disturbances following stress, frailty occurs, increasing the risk of adverse outcomes, such as falls, disability, hospitalization, and mortality (Hoogendijk et al., 2019). According to the vulnerability dimensions, frailty is further divided into physical, cognitive, social, and psychosocial phenotypes (Fried et al., 2001; Ruan et al., 2015; Yamada and Arai, 2018; Solfrizzi et al., 2019). At the system/organ level, metabolically active organs such as the brain, heart, and auditory system show higher vulnerability to given stressors, which results in diseases of these organs/systems, such as cognitive, psychosocial, cardiovascular, and hearing-related disorders.

Therefore, the vulnerability to chronic stresses might be the major cause of the phenotypic heterogeneity of ARHL and/or tinnitus, apart from age-related decline of functional reserve, internal environment (genetic determinants) and experience influence. However, the contribution of chronic non-audiogenic and audiogenic stresses to phenotypic heterogeneity of ARHL and/or tinnitus remains elusive. In the narrative review, our purpose is to bring insights regarding the phenotypic heterogeneity of presbycusis and subjective tinnitus in humans. We searched PubMed for articles published in English up to September 05, 2019, with the search terms “chronic stress,” “audiogenic stress or audiogenic (noise) impairment,” “vulnerability to chronic stress or audiogenic stress,” “heterogeneity of presbycusis and/or tinnitus,” “frailty or comorbidity and presbycusis and/or tinnitus.” We also sought publications from the reference lists of identified papers and from our cumulative literature archives. Moreover, we gave priority to systemic reviews and studies published in the past 10 years. Firstly, we compared the phenotypic heterogeneity of presbycusis and/or tinnitus after chronic non-audiogenic and audiogenic stresses exposure-induced systemic maladaptive reactions in older population, including frailty phenotypes and comorbidities. Then, we explore the mechanisms underlying heterogeneous presbycusis and/or tinnitus. Finally, the clinical implications of the phenotypic heterogeneity of presbycusis and/or tinnitus are discussed. An age-related integrative response model to cumulative stressors in the central auditory and limbic systems, and non-CNS or non-neuronal organs is suggested to understand the heterogeneity of ARHL and/or tinnitus.

## 2 The effect of chronic non-audiogenic stress-induced systemic vulnerability on heterogeneous presbycusis and/or tinnitus

**FIGURE 2:**
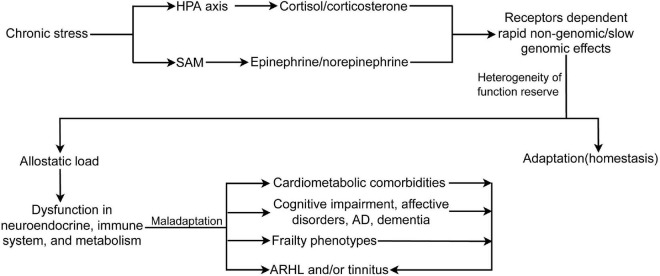
Chronic stress activates HPA axis and SAM, induces receptors dependent rapid non-genomic/slow genomic effects, and allostatic load, which causes dysfunction in neuroendocrine and immune system, and metabolic abnormality. In turn, allostatic load leads to different frailty phenotypes, comorbidities, and ARHL and/or tinnitus. Moreover, frailty phenotypes and comorbidities also can cause or exacerbate ARHL and/or tinnitus. HPA, hypothalamus-pituitary-adrenal axis; SAM, sympathetic adrenal–medullary axis; AD, Alzheimer disease; ARHL, age-related hearing loss.

Stress is referred to as an ongoing adaptive process of assessing the environment, coping with it, and enabling the individual to anticipate and deal with future challenges (McEwen and Akil, 2020). Stress mainly includes environmental (home and work), physiological (inflammation, metabolic syndrome, cardiovascular disease, and other diseases), and psychosocial (sleep problems, isolation, poverty, divorce, and abuse) components. Stress also encompasses demanding lifestyles, unhealthy diet, alcohol consumption, smoking, inadequate sleep, and physical inactivity. The recovery of functional reserve after short-term stress is an adaptive reaction that is generally beneficial to health. Highly heterogeneous non-chronic audiogenic stress, or cumulative stress exposure, is a common experience across time and results in different physical, cognitive, and psychosocial disorders. To face these stressful challenges, allostatic systems, including the neural, neuroendocrine, and neuroendocrine-immune systems, produce active and adaptive response processes that maintain homeostasis (McEwen, 1998; McEwen and Akil, 2020). Repeated or sustained activation of the allostatic systems by chronic non-audiogenic stress causes allostatic load/overload and maladaptive responses (Figure 2). The HPA axis and the SAM system are the two main components of allostasis that actively promote adaptation and homeostasis maintenance through mediators, such as cortisol (corticosterone in rodents) by HPA and two catecholamines, epinephrine and norepinephrine, by the SAM. Stress activates the release of CRH and arginine vasopressin from the PVN. These hormones stimulate the release of ACTH from the anterior pituitary, leading to the synthesis and release of glucocorticoids and mineralocorticoids (aldosterone) from the adrenal cortex. The physiological effects of glucocorticoids include metabolic regulation of carbohydrates, lipids, and proteins; immune regulation; and brain functions, such as emotion and cognition (Goncharova, 2020). In addition, glucocorticoids provide negative feedback at different levels, including neuroendocrine neurons in the PVD, pituitary gland, and higher brain centers (hippocampus, amygdala, and PFC) that are involved in the regulation of neuroendocrine neurons (Cullinan et al., 1993; Herman et al., 1995). These effects are regulated by the GR and MR. GR is widely expressed in different organs, while MR is selectively expressed in some organs, such as the brain (predominantly in the limbic structures, hippocampus and PFC, and hypothalamus), kidney, eye, and inner ear. MR has 10 times higher affinity for glucocorticoids than GR, which contributes to the biphasic and inverted U-shaped effects of glucocorticoids in response to stress (Rao and Androulakis, 2019). Glucocorticoids induced by stressors cause two types of stress reactions via GR and MR in target organs: rapid non-genomic and slow genomic reactions. The former is mediated by a minor membrane-bound GR or MR (McEwen and Akil, 2020), including direct stimulation of glutamate release and endocannabinoid secretion (Atsak et al., 2015), which then provides feedback on glutamate and GABA release and actions in mitochondria to affect Ca^2+^ buffering and free radical formation (McEwen et al., 2016; McEwen and Akil, 2020). GR and MR are ligand-dependent transcription factors mainly located in the cytoplasm. The binding of corticosteroids to GR or MR induces complex translocation to the nucleus, where the transcription of target genes is either enhanced or inhibited (Weikum et al., 2017). In addition to stress, MR has been implicated in the regulation of basal circadian rhythms and pulsatile patterns of glucocorticoids and ACTH (Gjerstad et al., 2018). MR controls the secretion of ACTH via mediating CRH release in the medial parvicellular neurons of the PVN by GABAergic neuron projections from the amygdala (Herman et al., 2016). When the MR-mediated negative feedback mechanism and the circadian rhythm are disrupted or blunted due to aging and chronic stress (Wilkinson et al., 2001; Goncharova, 2020), the induced impairment in the hippocampus or in the circuit of the hippocampus and PVD with a relay station in the bed nucleus of the stria terminalis (Cullinan et al., 1993) can cause persistent actions of stress hormones, allostatic load, and maladaptation in the brain and other physiological systems. Early life stress or experience also induces long-term hyperactivity of the HPA axis by epigenetic mechanisms, including DNA methylation of GR and CRH, and histone deacetylation (Garrido, 2011; Goncharova, 2020; Roesler et al., 2021) (Figure 2).

### 2.1 Vulnerability to chronic non-audiogenic stress, cognitive, and affective disorders

Individual differences in functional reserve/vulnerability (genes, development, and experience) of multiple physiological systems to allostatic load play an important role in the heterogeneity of adverse outcomes, including presbycusis and/or tinnitus. In the brain, the limbic structures, hippocampus, amygdala, and medial frontal cortex are the vulnerable interconnected regions, and structural plastic changes in one region may influence functional plasticity in other regions. Maladaptive plasticity of the hippocampus and PFC results in cognitive impairment, AD, and dementia (Popoli et al., 2011; Price and Duman, 2020). Hippocampal functional networks consist of the dorsal hippocampus (posterior hippocampus in primates), which performs cognitive functions, especially spatial processing, and the ventral hippocampus with stronger connectivity with the amygdala and hypothalamus, which are involved in processing emotion and stress. Acute stress leads to cytotoxicity and metabolic vulnerability in CA1 neurons owing to glucocorticoid-enhanced glutamatergic transmission with an increase in calcium influx. Regional vulnerability may result from genomic-dependent differences in the distribution of NMDA receptors, antioxidant enzymes, and inflammatory reactions (Bartsch and Wulff, 2015). Stress hormones have bidirectional effects on hippocampal plasticity. At low stress hormone levels, an increase in hormone levels facilitates LTP and consolidates memory, while at high stress hormone levels, there is a further increase in LTP and memory impairment (Tatomir et al., 2014). Increased glucocorticoid levels induced by chronic stress and aging cause hippocampal atrophy and dysfunction, including shrinkage of dendritic arborization in the CA3, NMDA-related excitotoxicity, BDNF decline, and LTP inhibition. Hippocampal damage further disrupts the inhibitory feedback on the HPA axis, resulting in increased glucocorticoid levels, which bind to the GR, causing a decline in neurogenesis in the dentate gyrus (Roozendaal et al., 2009).

The BLA, also known as the basolateral complex, is another vulnerable structure of the limbic system. BLA projections to the hypothalamus, bed nucleus of the stria terminalis, and midbrain via the central and medial amygdala regulate stress-induced autonomic, behavioral, and hormonal responses, while projections to the hippocampus, PFC, caudate nucleus, and nucleus accumbens via the entorhinal cortex pathway modulate learning and memory (Roozendaal et al., 2009). Chronic stress-induced high glucocorticoids cause a decline in GABAergic inhibition of the BLA and a rapid increase in glutamatergic plastic inputs, which cause widespread structural changes, dendritic elongation, and new spinogenesis in both primary and secondary dendrites. Meanwhile, the interconnected hippocampus and PFC concurrently produce reversible (less so in aged animals) retraction of dendrites, loss of synapses, and neurogenesis in the dentate gyrus. In other areas, such as the BLA and orbitofrontal cortex, stress leads to the expansion of dendrites and synaptogenesis (Vyas et al., 2002; Roozendaal et al., 2009; Chattarji et al., 2015; Lau et al., 2017; McEwen and Akil, 2020). Synaptic functional plasticity, such as glutamate neurotransmission in the PFC and hippocampus mediated by stress at pre (synthesis, transport, and release) and post-synaptic (glutamate recycling and receptors) stages, also produce adaptive changes (Popoli et al., 2011; Mazurek et al., 2012; McEwen et al., 2016). Dendritic hypertrophy of pyramidal neurons in the BLA is induced by rapid non-genomic responses, in which stress hormones are bound to GR or MR on the membrane, and by slow genomic responses. In genomic responses, the binding of glucocorticoids to cytosolic GR or MR translocates to the nucleus and increases the expression of proplasticity genes in the BLA (Reichardt, 2006) and decreases in the hippocampus and PFC (Duman et al., 2016), such as BDNF.

Repeated chronic stress results in hypertrophy of pyramidal neurons in the BLA, which fosters maladaptive neural plasticity in the hippocampus, contributing to the memory consolidation of negative emotional events, depression, and anxiety behaviors (Patel et al., 2018). Pyramidal neurons in the BLA complex of the amygdala and glucocorticoids from the HPA axis act synergistically with norepinephrine from the SAM to induce LTP and consolidation of long-term memory of emotionally arousing experiences (Quirarte et al., 1997; Atsak et al., 2015). In rapid non-genomic reactions, glucocorticoids induced by emotionally arousing events bind to membrane-bound GR, which activates the intracellular cAMP/PKA signaling pathway to induce endocannabinoid synthesis. Endocannabinoids bind endocannabinoid 1 receptors on GABAergic terminals and inhibit the release of GABA, which disinhibits norepinephrine release and increases the excitability of BLA pyramidal neurons and glucocorticoid effects on memory storage (Atsak et al., 2015).

Early life stress can increase brain vulnerability during aging. Maternal separation during early life increases cognitive deficits in aged mice (Solas et al., 2010). Epigenetic mechanisms may contribute in part to this effect. Prenatal exposure to maternal depression in humans (Oberlander et al., 2008), reduced maternal care in rats (Weaver et al., 2004), enhanced methylation of the GR gene in the promoter region, and reduced GR expression in the hippocampus induce long-term hyperactivity of the HPA axis and have adverse consequences (Goncharova, 2020). BLA activity can modulate the facilitation of memory consolidation through epigenetic mechanisms, such as DNA methylation and histone deacetylation, to enhance the transcription of memory-related genes in the hippocampus (Roesler et al., 2021). Efficient encoding of emotional memories by the amygdala can cause maladaptive long-term outcomes in psychological or mental diseases, such as depression and anxiety (Lau et al., 2017; Arnone, 2019; Duman et al., 2019). Correspondingly, glucocorticoid- and noradrenaline-induced activation of the BLA may modulate memory processes in other brain areas, such as the hippocampus and PFC, and impair cognitive skills such as memory and executive function.

### 2.2 Vulnerability to chronic non-audiogenic stress and frailty phenotypes and comorbidities

Allostatic load induced by chronic stress can cause metabolic vulnerability. Stress increases energy consumption to meet the demands of behavioral responses, physiological changes, and cellular functions. Glucocorticoids mobilize energetic substrates, including glucose, lipids, and amino acids, into the circulation via genomic and non-genomic effects (Picard et al., 2018). Mitochondria in metabolically active organs, such as the brain and heart, transform these substrates and oxygen into ATP to provide energy for the maintenance of all cellular and organ functions, as well as the synthesis of hormones and catecholamines during periods of stress to ensure adequate circulating levels of these energy substrates (Picard et al., 2018). Individuals with higher circulating levels of cortisol can develop metabolic dysfunction, insulin resistance, and a prediabetic state (Phillips et al., 1998). Mice exhibit physical inactivity and depression-like behavior after chronic corticosterone administration (Karatsoreos et al., 2010). Chronic stress accumulation and early life adverse experiences, such as abuse and neglect, induce hyperactivity of the HPA axis by epigenetic mechanisms, significantly increasing the risk of diabetes, cardiovascular disease, and mental health problems, including depression and later dementia (Shonkoff et al., 2009; Miller and Lumeng, 2018).

In later life, the aerobic reserve markedly decreases, and anaerobic pathways must be available to meet most of the daily basic task challenges (Waters et al., 1988). Sustained anaerobic metabolism triggers fatigue and an age-related decline in physical activity. Aging and chronic stress-induced decrease in skeletal muscle mass and/or activation of the CNS further cause physical fatigue, exhaustion or weariness, and sedentary behavior (Evans and Lambert, 2007), which ultimately lead to physical and other frailty phenotypes.

Allostatic load induced by chronic stress can cause immune system dysfunction via the HPA and SAM axes. Glucocorticoids bind to GRs on the outer membrane, causing impaired lymphocyte function (Kim et al., 2016). Peripheral monocytes and macrophages and central microglia and astrocytes respond to catecholamines from the adrenal medulla by adrenoceptors, increasing the release of proinflammatory mediators, including IL-1, IL-6, and tumor necrosis factor-α (Kim et al., 2016; Lee and Giuliani, 2019). The release of the anti-inflammatory cytokine IL-10 from the immune cells of the thymus gland and bone marrow is decreased, which is mediated by noradrenergic afferent innervations (Kim et al., 2016; Lee and Giuliani, 2019). Meanwhile, chronic neuroinflammation inhibits GR function, which causes GR functional resistance and HPA axis activation and further exacerbates proinflammatory cytokine activity and brain impairment (Kim et al., 2016). Maladaptive responses of microglia may exacerbate pathological damage by chronic stress via dysregulated complement-mediated phagocytosis, release of inflammatory cytokines, mediation of oxidative stress, and decreased ability to phagocytose accumulated amyloid beta (Aβ) and pathological tau in AD (Bisht et al., 2018).

### 2.3 Vulnerability to chronic non-audiogenic stress and phenotypic heterogeneity of ARHL and tinnitus

Chronic stress, low-grade systemic inflammation (Marsland et al., 2008), and metabolic dysfunctions, such as metabolic syndrome (Yau et al., 2012), not only induce brain (especially hippocampal shrinkage and maladaptive alterations in cognition, including episodic, semantic, and spatial memory), neuroendocrine, and psychiatric vulnerability, but also cause or aggravate ARHL and tinnitus together with life experiences and aging (Mazurek et al., 2012; Canlon et al., 2013; Henry et al., 2014; Watson et al., 2017; Seicol et al., 2022). The hippocampus interconnects directly or indirectly with the auditory cortex via the parahippocampus or the perirhinal cortex. A major function of the hippocampal-auditory system is to promote storage of long-term auditory memory in the neocortex. The dorsal hippocampus (posterior hippocampus in primates) primarily performs cognitive functions, such as auditory information processing. Alpha-bungarotoxin-sensitive nicotinic receptors on hippocampal GABAergic neurons in rats have been reported to mediate auditory sensory gating, wherein the hippocampus rapidly inhibits its response to repetitive auditory stimulation (Adams and Stevens, 1998; Adams et al., 2000). Hippocampal emotional processing may play a crucial role in the learning and storage of aversive memories. Moreover, the hippocampus, interconnected amygdala, and PFC can affect auditory processing (Kraus and Canlon, 2012). The amygdala mediates auditory activity and plasticity of fear conditioning and augments the receptive fields for frequencies of the unconditioned emotional valence auditory stimuli via cholinergic projections to activate both the nucleus basalis PFC and auditory cortex (Froemke and Martins, 2011), which might be involved in aversive tinnitus long-term memory. Amygdalar neurons also directly project to the inferior colliculus, which is involved in emotion-mediated regulation of auditory stimulus processing (Marsh et al., 2002).

Various epidemiological studies have shown that immune dysfunction (chronic systemic inflammation, rheumatoid arthritis, systemic lupus erythematosus, and sclerosis), endocrine metabolic diseases (diabetes mellitus, hyperinsulinemia, hypothyroidism, atherosclerosis, hypertension, and stroke), neurological diseases (meningitis, migraine, epilepsy, and brain trauma), lifestyle factors (smoking and alcohol consumption), and psychological stress (sleeping problems, depression, and anxiety) are risk factors for ARHL and/or tinnitus (Baguley et al., 2013; Knipper et al., 2013; Panza et al., 2015; Jafari et al., 2019; Haider et al., 2020; Simoes et al., 2021).

## 3 Chronic audiogenic stress and phenotypic heterogeneity of ARHL with tinnitus

### 3.1 The phenotypic heterogeneity of ARHL with tinnitus

In addition to the above-mentioned risk factors, including genetic predisposition and experiences (auditory reserve), chronic audiogenic stresses include auditory aging, noise exposure, and otological diseases such as otosclerosis, vestibular schwannoma, Ménière’s disease, infectious diseases, and ototoxic medications. These different stressors cause different pathophysiological alterations of ARHL, such as cochlear conductive, sensory, neural, metabolic or strial, mixed, and indeterminate (Gates and Mills, 2005). Some forms of ARHL are not associated with a deficit of the peripheral auditory system but with CAP dysfunction (Panza et al., 2015). Individuals with different pathological phenotypes exhibit heterogeneous clinical manifestations. For instance, individuals with CAP dysfunction can understand speech in a quiet environment but have great difficulty in understanding speech in an environment with background noise. Individuals with hidden hearing loss due to ribbon synaptopathy between IHCs and afferent auditory nerve fibers indicate a normal hearing threshold in audiograms by standard audiological assessment (Kujawa and Liberman, 2009, 2015; Shore et al., 2016; Liberman and Kujawa, 2017). According to the accompanying comorbidities, ARHL can be classified into different phenotypes, such as cognitive impairment and psychological disorders (Gates and Mills, 2005; Panza et al., 2015; Wayne and Johnsrude, 2015; Jafari et al., 2019). Chronic subjective tinnitus refers to phantom perception in the absence of an external acoustic source. According to the common neuropathological and psychopathological comorbidities, tinnitus is classified into several phenotypes. Tinnitus accompanies auditory function decline at different degrees. Noise induced hearing loss and ARHL are common comorbidities (Baguley et al., 2013; Knipper et al., 2013; Jafari et al., 2019). Hyperacusis, referred to as a pretinnitus state, usually coexists with auditory dysfunction and tinnitus (Jastreboff and Hazell, 1993). Bothersome tinnitus accompanying psychological disorders such as depression and anxiety are also common phenotypes (Langguth et al., 2011; Pattyn et al., 2016; Jafari et al., 2019). Another tinnitus phenotype involves interactions with the somatosensory system, including somatosensory-related or -mediated tinnitus (Shore et al., 2007).

The co-occurrence of different frailty phenotypes usually contributes to the heterogeneity of ARHL and/or tinnitus in older adults (Ruan et al., 2021), resulting in a lower quality of life (Joo et al., 2015; Zhang W. et al., 2021). ARHL is associated with frailty, as assessed using the FRAIL scale and frailty index (Tian et al., 2022). Compared with older adults with normal hearing, those with moderate or greater hearing impairment had a 63% increased risk of physical frailty and a greater annual percent increase in fall occurrence (Kamil et al., 2016). In older adults, hearing loss was associated with postural instability (Bang et al., 2020), disability or limitation of physical function (Chen et al., 2014), and frailty syndrome (Yévenes-Briones et al., 2021). Individuals with moderate ARHL had an odds ratio of 2.24 for social frailty compared to those with normal hearing after adjusting for confounding factors, including physical frailty (Yoo et al., 2019). Longitudinal data have shown that comorbid hearing problems and social frailty are the largest risk factors for disability (Bae et al., 2018a). For instance, longitudinal and cross-sectional studies have shown that social frailty (including loneliness and social isolation) aggravates ARHL-related episodic amnesia (Maharani et al., 2019; Loughrey et al., 2021) and multiple-domain mild cognitive impairment (Bae et al., 2018b). Loneliness and social isolation due to communication difficulties may also contribute to psychological disorders or frailty in individuals with ARHL. Individuals with cognitive impairment or frailty had a higher risk of severe ARHL/tinnitus and comorbid ARHL/tinnitus than those with physical frailty. Furthermore, individuals with reversible cognitive frailty have a higher probability of having less ARHL/tinnitus and comorbid ARHL/tinnitus than those with potentially reversible cognitive frailty (Ruan et al., 2021).

### 3.2 The potential mechanism of ARHL with tinnitus heterogeneity induced by chronic audiogenic stress

**FIGURE 3:**
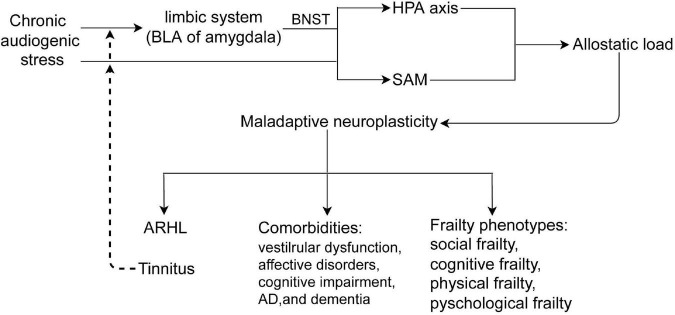
Chronic audiogenic stress activates HPA axis by limbic system-BNST and SAM, induces allostatic load, which causes maladaptive neuroplasticity that contributes to ARHL and/or tinnitus, comorbidities, and frailty phenotypes. Tinnitus, a internal audiogenic stressor, could further activates HPA axis and SAM in a vicious circle. BLA, the basolateral nucleus of amygdala; BNST, the bed nucleus of stria terminalis; HPA, hypothalamus -pituitary-adrenal axis; SAM, sympathetic adrenal–medullary axis; ARHL, age-related hearing loss.

The pathophysiology of ARHL and/or tinnitus is complex and multifactorial, involving adaptive and maladaptive neuroplasticity of the auditory and non-auditory systems, especially the limbic system, which is involved in negative emotion memory. ARHL and/or tinnitus are also closely associated with cognitive impairment, psychological disorders, and frailty. Although the precise pathophysiology of ARHL and/or tinnitus, as well as the heterogeneous effects on the above comorbidities, remains elusive, differences in the auditory and non-auditory systems vulnerability to chronic stress, and maladaptive neuroplasticity to allostatic load might be potential mechanisms (Figure 3).

#### 3.2.1 Allostatic load and maladaptive auditory pathway neuroplasticity

Apart from the indirect effects of allostatic load on non-auditory systems, chronic stress can directly induce cochlear damage and neuroplasticity in the central auditory system. The accumulation of chronic mixed stress exposure, including to physical and psychosocial stressors such as noise exposure, ototoxic drugs, and sleeping problems, is an important cause of ARHL with tinnitus. Long-term stress exposure, such as occupational or recreational noise, significantly increases the risk of hearing impairment and tinnitus (Baigi et al., 2011; Canlon et al., 2013; Jafari et al., 2022). Animal studies have indicated a direct interconnection between the thalamine auditory nucleus and PVN (Campeau and Watson, 2000). A lesion study in rats indicated that the removal of the MGB (auditory thalamus) blocks the HPA axis-mediated stress hormones, corticosterone release, and c-fos mRNA induction (which is upregulated in neurons with increased activity) in the medial parvocellular neurons of the paraventricular hypothalamic nucleus (Campeau and Watson, 2000). Both the HPA axis and SAM have been reported to cause adaptive reactions to acute stress in the auditory system. The MR and GR of stress hormones are expressed in the inner ears of humans and experimental animals (Canlon et al., 2007; Mazurek et al., 2012). In particular, MR mRNA is expressed at the highest level in the inner ear (Thomas and Harvey, 2011). Moreover, the MR is mainly located in the marginal cells of the stria vascularis (Furuta et al., 1994). GR- and MR-mediated genomic reactions may decrease inner ear damage and increase hearing sensitivity to acute acoustic stress, such as noise exposure and ototoxic drugs. The GR- or MR-corticosteroids complex translocates into the nucleus and then GR or MR bind to the glucocorticoid response element to regulate gene expression or down-stream transcription factors, such as NF-κB (Canlon et al., 2007; Mazurek et al., 2012). Glucocorticoid-inducible kinase 1 is involved in GR- and MR-mediated, rapid non-genomic reactions and is expressed in the inner ear structures of experimental animals, including the stria vascularis, spiral ligament, spiral limbus, organ of Corti, Reissner’s membrane, and spiral ganglion (Zhong and Liu, 2009). The SAM axis also participates in cochlear stress reactions. Non-perivascular sympathetic fibers from the superior cervical ganglion can modulate afferent auditory signals and decrease the vulnerability of experimental animals to acute noise exposure (Hildesheimer et al., 1991; Bielefeld and Henderson, 2007).

The heterogeneity of stressors, such as noise exposure, aging, ototoxic drugs, strength and duration of stress, and differences in peripheral auditory structural vulnerability to stressors and allostatic load can cause maladaptive reactions with various cochlear pathological changes. The most vulnerable cochlear structure might be the synapses between the modiolar side of IHCs with a low spontaneous discharge rate and high-threshold type I afferent fibers (Liberman, 1978). The selectively low spontaneous rate of synaptic damage induced by noise exposure or aging is referred to as “hidden hearing loss” (Kujawa and Liberman, 2009, 2015). These mechanisms include glutamate excitotoxicity via NMDA receptors and maladaptive responses, such as a decrease in the number of presynaptic ribbons and paired pre- and postsynaptic structures (Kujawa and Liberman, 2009; Rüttiger et al., 2013) and disruption of the first heminodes at the auditory nerve peripheral terminal due to the transient loss of Schwann cells (Wan and Corfas, 2017). The fibrocytes in the spiral ligament, particularly type IV cells, were vulnerable in C57BL/6 mice with ARHL. Degeneration of fibrocytes precedes the loss of hair cells and spiral ganglions (Hequembourg and Liberman, 2001). Among sensory cells, outer hair cells are more vulnerable to stressors than IHCs, and the loss of hair cells starts in the high-frequency base turn. Stress causes improper potassium-sodium balance in the endolymph and external lymph in the scala tympani due to cell dysfunction in the stria vascularis and outer hair cells. Noise exposure induces the decline in mechano-electrical transduction channel opening due to stereocilia bundle damage of outer hair cells and the increase in endocochlear potential (Patuzzi, 2002), which can depolarize IHCs, in turn, an intracellular influx of Ca^2+^ mediated by voltage-gated Ca^2+^ channels, and glutamate release from the presynaptic ribbon, which activates Type I afferent fibers. Excessive glutamate release causes excitotoxicity in afferent fibers via NMDA receptors. The energy requirement ATP to maintain the potassium-sodium balance in the endolymph results in excessive production of reactive oxygen or hydrogen species, which also contributes to the damage of cochlear structures such as hair cells, spiral ganglion, and cells in the stria vascularis. Indeed, antioxidants can counteract noise-induced cochlear redox imbalance and alterations in the dendritic architecture of pyramidal neurons in the auditory cortex (Fetoni et al., 2015). All types of cochlear maladaptive pathological changes due to exposure to cochlear stressors cause reduced auditory nerve activity (input to the central auditory system), which indicates a reduction in the amplitude of the acoustic nerve CAP.

Peripheral deafferentation induced by aging and other audiogenic stresses results in allostatic load and adaptive/maladaptive responses, such as increased SFR and temporal synchrony in the firing pattern of neurons in the central auditory system, which attempt to restore homeostasis in response to plastic compensatory changes in central auditory structures (Baguley et al., 2013; Knipper et al., 2013; Gröschel et al., 2014; Henry et al., 2014; Shore et al., 2016; Simoes et al., 2021). One of the structural plastic changes is the disinhibition of neurons in the central auditory system, especially in the cochlear nucleus (dorsal cochlear nucleus, DCN; ventral cochlear nucleus, VCN) and the inferior colliculus. Decreased input within the damaged peripheral auditory region downregulates inhibitory neurotransmitters and corresponding receptors, including glycine and GABA, in both the DCN and VCN (Shore et al., 2007; Middleton et al., 2011; Wang et al., 2011; Gröschel et al., 2014), and GABA in the inferior colliculus (Bauer et al., 2008; Wang et al., 2011). There was also an increase in excitatory neurotransmission in the central auditory system. In addition to the decline in afferent input, the vulnerability increase in inhibitory neurons with high energy demands that leads to oxidative damage might contribute to the downregulation of inhibition in aging animals (Ibrahim and Llano, 2019). Another structural plastic change is tonotopic map reorganization. Cochlear input reduction at a given frequency causes neurons within the primary auditory cortex to respond to the characteristic frequency that responds to adjacent frequencies at the edge of hearing loss (Engineer et al., 2011).

Although the time course of SFR presence and the edge of hearing loss do not correspond to tinnitus perception and pitch, respectively, maladaptive neuroplasticity, both through disinhibition and tonotopic map reorganization, modify the gain (hyperexcitability) of central neurons, including elevated SFR, enhanced bust firing, and synchronicity, which might be involved in the generation of hyperacusis and tinnitus. Maladaptation of the intrinsic membrane properties of central neurons might play a critical role. Clinical studies have provided evidence that subjects with hidden hearing loss and tinnitus exhibit decreased auditory neural activity and increased central gain. The wave I amplitude of the auditory brain response to suprathreshold sounds is reduced, and the wave V amplitude is normal or enhanced in individuals with tinnitus (Schaette and McAlpine, 2011; Gu et al., 2012). Animal experiments on noise-induced hidden hearing loss indicate that animals with tinnitus show more LTP and lower long-term depression than animals without tinnitus (Koehler and Shore, 2013). Animals with tinnitus demonstrate increased SFR or bursting in the cochlear nucleus and MGB (Kalappa et al., 2014; Wu et al., 2016). MGB neurons from animals with tinnitus fired more spikes per burst than non-tinnitus MGB neurons (Sametsky et al., 2015). Compared with those without tinnitus, animals with noise-induced hearing loss and tinnitus showed an increased SFR of fusiform cells in the DCN with increased vulnerability of KCNQ2/3 and/or HCN channels (Li et al., 2015), and synchronous activity of neurons in the IC (increased wave V amplitude at suprathreshold levels), accompanied by a reduction in the levels of activity-regulated cytoskeletal proteins in the auditory cortex (Singer et al., 2013).

#### 3.2.2 Maladaptive neuroplasticity in extra-auditory brain regions

In addition to intramodal plasticity in the central auditory pathway, crossmodal plasticity also occurs when the vulnerable neural structures with deprived function (hearing loss) are recruited by intact sensory modalities, such as visional or somatosensory systems, for visual and somatosensory processing. Auditory deafferentation is a critical triggering factor that initiates maladaptive crossmodal plastic reorganization, which may cause or modulate tinnitus. The cochlear nucleus is the first relay of the central auditory system and a site for the integration of multimodal information. Somatosensory inputs from the dorsal root and trigeminal ganglia can directly and indirectly innervate the DCN, VCN, and inferior colliculus. The cochlear nucleus receives auditory afferences associated with VGLUT 1 distributions and other projections with VGLUT2 distributions, such as somatosensory and vestibular afferent fibers (Barker et al., 2012; Heeringa et al., 2016). After unilateral deafferentation induced by noise exposure or ototoxic drugs, VGLUT 1 expression was significantly increased in the ipsilateral CN, but VGLUT2 expression was upregulated in the fusiform cell layer of the DCN, granule cell lamina, and anteroventral cochlear nucleus, which are known to receive somatosensory terminals (Heeringa et al., 2016, 2018). The lateral vestibular nucleus (LVN) innervates the bilateral fusiform and deep layers of the DCN with VGLUT2 distribution. Noise exposure results in a compensatory upregulation of the input from VGLUT2-mediated projections from the LVN to the DCN and a downregulation of the input from VGLUT 1-mediated auditory afferentation (Barker et al., 2012). A rare tinnitus phenotype in the deaf ear on the side of the surgery, modulated by peripheral eye gaze, has been reported after vestibular schwannoma removal. Maladaptive plastic reorganization, including neuronal sprouting from the para-abducens nucleus, abducens nucleus, or medial longitudinal fasciculus to the CN, and from the trigeminal nerve with somatosensory projections to the CN, might contribute to tinnitus generation and regulation (van Gendt et al., 2012). In summary, deafness induced by noise exposure, aging, and ototoxic drugs is a critical trigger. The increased SFR and LPT of the DCN fusiform neurons that receive non-auditory excitatory projections might be the cause of tinnitus onset. Conscious perception and maintenance of tinnitus requires long-range connectivity between auditory and non-auditory regions (De Ridder et al., 2011; Langguth et al., 2012). Both self-awareness and salience brain networks contribute to tinnitus perception, and the distress network that contains the anterior cingulate cortex, anterior insula, and amygdala contributes to negative emotional responses associated with tinnitus severity. Memory networks consisting of the hippocampus and parahippocampus might be involved in the persistence of tinnitus phantom perception and negative emotions, such as fear-motivated memory. However, distress behavior may be a comorbidity of tinnitus associated with stressors. Noise exposure, a psychological stressor, not only causes hearing loss with tinnitus but also directly activates the limbic-HPA-axis and triggers negative emotional reactions.

The ARHL and/or tinnitus can also be considered internal stressors that cause allostatic load and maladaptive neuroplasticity in extra-auditory brain regions. An exemplary study of long-term chronic tinnitus indicated that subjects with high tinnitus-related distress had greater increases in chronic cortisol levels and intolerance to external sounds than those with low tinnitus-related distress and control subjects (Hébert et al., 2004). Distressed individuals with tinnitus were reported that indicate a blunted cortisol response in a cross-control study (Jackson, 2019). In this study, salivary cortisol awakening response was assessed in tinnitus and non-tinnitus participants. Tinnitus participants were divided into two groups based on tinnitus severity. Saliva samples were collected at awakening and at 4–5 times after awakening [0 min (wakening), +15 min, +30 min, +45 min, +6 h and +12 h). A significant main effect of group was found that area under the curve with respect to increase levels were significantly lower in the high tinnitus severity group (distress tinnitus) than in control or low tinnitus severity group. The vestibular system is anatomically and functionally connected to the cochlea in the inner ear, and both are regulated during stress responses. Compared to vestibular cross-modal compensatory plasticity after hearing stress, vestibular damage is more common (Szczepek and Stankovic, 2021). The high expression of stress MRs in the stria vascularis might result in maladaptation, including decreased spontaneous cochlear activity due to the decline of the endocochlear potential, and sodium and potassium imbalance in both the scala tympani and vestibuli. In turn, it causes comorbidities of vestibular dysfunction such as abnormal balance and spatial orientation.

Audiogenic stresses can cause allostatic loads in cognitive- and emotion-related limbic systems, as well as maladaptive plasticity, both structurally and functionally. ARHL mice show synaptic degeneration in the hippocampus accompanied by cognitive impairment, which is closely associated with the severity of hearing loss (Yu et al., 2011). Clinical studies have shown that ARHL is associated with higher cerebrospinal fluid levels of tau or ptau181, atrophy of the hippocampus and entorhinal cortex (Xu et al., 2019), and a higher Braak stage (increased neurofibrillary tangle burden) (Brenowitz et al., 2020; Lozupone et al., 2020). These associations were more significant among cognitively unimpaired/non-dementia-stage subjects at baseline. Sensorineural hearing loss induced by ototoxic drugs in experimental animals also causes cognitive impairment, inhibition of neurogenesis, increased tau protein hyperphosphorylation, and neuroinflammation in the hippocampus (Shen et al., 2021). Chronic noise exposure causes both cognitive impairment and hearing loss along with tinnitus, which is associated with long-lasting activation of the HPA axis (Gai et al., 2017). The contribution of both direct and indirect activation of the HPA axis to noise-induced hearing loss requires further exploration. Chronic noise exposure can cause spatial memory deficits, lasting tau hyperphosphorylation and neurofibrillary tangles in the rat hippocampus and PFC, and neurogenesis (Cui et al., 2012; Manohar et al., 2022).

Besides cognitive impairment and detrimental memory, chronic audiogenic stress also causes paralleled emotional responses and affective disorders. Animal studies have indicated that the maladaptive neuroplasticity of glutamatergic, GABAergic, and cholinergic inputs may regulate tinnitus-related fear-motivated memory in the hippocampus (Zhang et al., 2019; Zhang L. et al., 2021; Zhang W. et al., 2021). The maladaptive neuroplasticity of the hippocampus is mediated by autonomic/neuroendocrine activation via the BLA-HPA axis. The amygdala is the center of the salience network and is associated with attentional processing of emotional memory. Chronic audiogenic stress and internal chronic tinnitus stress may be similar to other chronic physical and psychosocial stressors that lead to structural and functional maladaptive plasticity in both the amygdala and hippocampus. These alterations include emotional memory enhancement due to amygdala hyperexcitability, impairment of spatial and declarative memory paralleled by dendritic hypertrophy in the amygdala, and dendritic atrophy and reduced neurogenesis in the hippocampus (Roozendaal et al., 2009; McEwen et al., 2016).

Chronic audiogenic stress (including aversive tinnitus) allows for emotion-induced enhancement of memory consolidation via amygdala-induced facilitation of hippocampal LTP (Abe, 2001). This mechanism might be involved in the chronic stress-induced activation of the BLA-HPA axis and SAM. The interaction of rapid catecholamine and non-genomic glucocorticoid effects in the BLA complex causes LTP of pyramidal neurons in the lateral amygdala, which facilitates emotional memory formation and suppresses other aspects of cognitive performance at the early stage of chronic stress through a synergistic action with the hippocampus. When slow genomic glucocorticoid actions become active, emotional memory consolidation and long-term memory storage are promoted by reducing interference through suppression of new information coding (Schwabe et al., 2012). An individual’s previous experience and stressful lifestyle can modify aversive emotional memory through an epigenetic mechanism. Chronic aversive tinnitus and ARHL lead to adverse consequences, including different frailty phenotypes, such as physical, cognitive, and psychosocial frailty, and comorbidities, such as fatigue, sleep disturbances, cardiometabolic disorders, cognitive and affective disorders.

## 4 Implications for the management of heterogeneous presbycusis and/or tinnitus

The phenotypic heterogeneity of ARHL and/or tinnitus in the elderly population is characterized by age-related decline in the functional reserve of multiple physiological systems. Although both chronic non-audiogenic and audiogenic stresses cause different phenotypes of ARHL and/or tinnitus, including frailty phenotypes, physical, cognitive, and affective comorbidities, the chronic non-audiogenic stresses usually lead to systemic abnormal adaption, and frailty phenotypes and physical comorbidities are more common, with milder ARHL and/or tinnitus. The chronic audiogenic stresses cause more severe ARHL and/or tinnitus, social frailty phenotype, and brain disorders with less physical comorbidities. Chronic audiogenic stresses also might result in different phenotypes of ARHL and/or tinnitus. In particular, cumulative noise exposure from the environment, traffic, and leisure activities, which is not only audiogenic stressor, but also is chronic psychosocial stressor. Cumulative noise exposure is the most common causes of ARHL with tinnitus (Baigi et al., 2011; Hasson et al., 2011; Jafari et al., 2020a), but also is susceptible to chronic bothered tinnitus, cognitive impairment (Chen et al., 2017; Jafari et al., 2020b), and emotional disorders (Jafari et al., 2022). The chronic aversive tinnitus, an interior audiogenic stressor, further exacerbates affective disorders.

The mechanisms induced by chronic stress underlying the phenotypic heterogeneity of ARHL and/or tinnitus, especially ARHL with tinnitus, remain unclear. Chronic stress or the cumulative different stressors throughout the lifespan induce abnormal activation of the HPA-axis and SAM, allostatic load in vulnerable organs, and maladaptive reactions, which constitute the main causes of the phenotypic heterogeneity of ARHL and/or tinnitus. Genetic factors, early life experiences, physical and social environment, and lifestyle not only modify the trajectory of functional reserve of the brain and body, but also affect rapid non-genomic and slow genomic effects mediated by stress hormones and receptors. Compared with chronic non-audiogenic stress, audiogenic stress might mainly activate HPA axis via limbic system, especially BLA. Popular hypotheses about mechanisms of ARHL and/or tinnitus include auditory deafferentation and central disinhibition (bottom-up), and reorganization of key neural networks responsible for attention, emotion, and audition (top-down) auditory attention. An age-related integrative response model to cumulative stressors, including chronic non-audiogenic and audiogenic stressors in the central auditory and limbic systems, and non-CNS or non-neuronal organs is required to understand the heterogeneity of ARHL and/or tinnitus.

Several systematic reviews had indicated that there are some potential biomarkers for diagnosis of subjective tinnitus. Late auditory evoked potential component, P300 could differentiate subjective tinnitus patients from controls (Cardon et al., 2020). A unit increase in the mean ratio V/I of auditory brainstem response will enhance 10% likelihood for moderate tinnitus (Haider et al., 2022). Some molecules from oxidative stress, inflammation, steroids, and neurotransmitters might be tinnitus biomarkers (Haider et al., 2021). Based on integrative response model to cumulative stressors, the omics-based technologies, such as proteomics, metabolomics, and epigenetics with good design and validation in a large old population cohort will be a new frontier to explore novel biomarkers and potential molecular mechanisms for the phenotypic heterogeneity of ARHL and/or tinnitus. The integrative model also suggests that there are common interventions and phenotypically specific interventions. The common interventions include the life style interventions and behavioral interventions, such as physical activity over the life course, especially during a critical developmental window, can improve functional reserve, decrease chronic stress-induced maladaptive reactions, and achieve disproportionately positive influences on health aging (McEwen and Akil, 2020). In the auditory system, early auditory experiences, such as music and foreign language training, can significantly improve auditory reserve (Skoe and Kraus, 2014; Kroll et al., 2015; Krizman et al., 2016). In contrast, noise exposure during the developmental critical window causes a significantly negative trajectory of auditory reserve and increases vulnerability to stressors in later stages (Jafari et al., 2020b). Phenotypically specific interventions are for the specific phenotypes of ARHL and/or tinnitus. Bimodal auditory somatosensory stimulation based on cross-modal plasticity and non-invasive brain stimulation attenuates tinnitus in humans (Marks et al., 2018; Chen et al., 2020). However, in some patients with emotional reactions to internal tinnitus sounds, treatment targeted at the auditory percept, or tinnitus are not effective. Cognitive behavioral therapies might be effective in treating ARHL with aversive tinnitus (Piccirillo et al., 2020). This phenotype may be involved in the long-term memory of aversive internal tinnitus sounds. Initiating and maintaining the suppression of long-term aversive tinnitus memory is a promising intervention.

In summary, chronic non-audiogenic and audiogenic stressors might activate HAP axis via different pathway, and cause different phenotypes of ARHL and/or tinnitus due to different vulnerability of physiological systems to stress responses. It is essential to understand the mechanisms that result in the phenotypic heterogeneity of ARHL and/or tinnitus as the basis for correct diagnosis and treatment. New predictive biomarkers and precision interventions for different phenotypes of ARHL and/or tinnitus are possible only when new insights into these mechanisms are achieved. Based on integrative model of stress responses, a primary principle for the management of ARHL and/or tinnitus is person-centered integrated care with the support of a multidisciplinary team, including audiologists, hearing therapists, psychologists, physicians, and geriatrists.

## Author contributions

QR: conceptualization and writing – review and editing. JR: writing – original draft. XH: illustrations and editing. YL and ZH: review and editing. All authors read and agreed to the published version of the manuscript.

## References

[B1] Abe K. (2001). Modulation of hippocampal long-term potentiation by the amygdala: A synaptic mechanism linking emotion and memory. *Jpn. J. Pharmacol.* 86 18–22. 10.1254/jjp.86.18 11430468

[B2] Adams C. E. Stevens K. E. (1998). Inhibition of nitric oxide synthase disrupts inhibitory gating of auditory responses in rat hippocampus. *J. Pharmacol. Exp. Ther.* 287 760–765. 9808707

[B3] Adams C. E. Stevens K. E. Kem W. R. Freedman R. (2000). Inhibition of nitric oxide synthase prevents alpha 7 nicotinic receptor-mediated restoration of inhibitory auditory gating in rat hippocampus. *Brain Res.* 877 235–244. 10986337 10.1016/s0006-8993(00)02677-9

[B4] Arnone D. (2019). Functional MRI findings, pharmacological treatment in major depression and clinical response. *Prog. Neuropsychopharmacol. Biol. Psychiatry* 91 28–37. 10.1016/j.pnpbp.2018.08.004 30099082

[B5] Atamna H. Tenore A. Lui F. Dhahbi J. M. (2018). Organ reserve, excess metabolic capacity, and aging. *Biogerontology* 19 171–184. 10.1007/s10522-018-9746-8 29335816 PMC5835208

[B6] Atsak P. Hauer D. Campolongo P. Schelling G. Fornari R. V. Roozendaal B. (2015). Endocannabinoid signaling within the basolateral amygdala integrates multiple stress hormone effects on memory consolidation. *Neuropsychopharmacology* 40 1485–1494. 10.1038/npp.2014.334 25547713 PMC4397407

[B7] Bae S. Lee S. Lee S. Harada K. Makizako H. Park H. (2018a). Combined effect of self-reported hearing problems and level of social activities on the risk of disability in Japanese older adults: A population-based longitudinal study. *Maturitas* 115 51–55. 10.1016/j.maturitas.2018.06.008 30049347

[B8] Bae S. Lee S. Lee S. Jung S. Makino K. Park H. (2018b). The role of social frailty in explaining the association between hearing problems and mild cognitive impairment in older adults. *Arch. Gerontol. Geriatr.* 78 45–50. 10.1016/j.archger.2018.05.025 29890382

[B9] Baguley D. McFerran D. Hall D. (2013). Tinnitus. *Lancet* 382 1600–1607. 10.1016/S0140-6736(13)60142-7 23827090

[B10] Baigi A. Oden A. Almlid-Larsen V. Barrenäs M. L. Holgers K. M. (2011). Tinnitus in the general population with a focus on noise and stress: A public health study. *Ear Hear.* 32 787–789. 10.1097/AUD.0b013e31822229bd 21716113

[B11] Bang S. H. Jeon J. M. Lee J. G. Choi J. Song J. J. Chae S. W. (2020). Association between hearing loss and postural instability in older Korean adults. *JAMA Otolaryngol. Head Neck Surg.* 146 530–534. 10.1001/jamaoto.2020.0293 32324231 PMC7180733

[B12] Barker M. Solinski H. J. Hashimoto H. Tagoe T. Pilati N. Hamann M. (2012). Acoustic overexposure increases the expression of VGLUT-2 mediated projections from the lateral vestibular nucleus to the dorsal cochlear nucleus. *PLoS One* 7:e35955. 10.1371/journal.pone.0035955 22570693 PMC3343051

[B13] Bartsch T. Wulff P. (2015). The hippocampus in aging and disease: From plasticity to vulnerability. *Neuroscience* 309 1–16. 10.1016/j.neuroscience.2015.07.084 26241337

[B14] Bauer C. A. Turner J. G. Caspary D. M. Myers K. S. Brozoski T. J. (2008). Tinnitus and inferior colliculus activity in chinchillas related to three distinct patterns of cochlear trauma. *J. Neurosci. Res.* 86 2564–2578. 10.1002/jnr.21699 18438941 PMC2614665

[B15] Ben-Shlomo Y. Cooper R. Kuh D. (2016). The last two decades of life course epidemiology, and its relevance for research on ageing. *Int. J. Epidemiol.* 45 973–988. 10.1093/ije/dyw096 27880685 PMC5841628

[B16] Bielefeld E. C. Henderson D. (2007). Influence of sympathetic fibers on noise-induced hearing loss in the chinchilla. *Hear. Res.* 223 11–19. 10.1016/j.heares.2006.09.010 17092669

[B17] Bisht K. Sharma K. Tremblay M. È. (2018). Chronic stress as a risk factor for Alzheimer’s disease: Roles of microglia-mediated synaptic remodeling, inflammation, and oxidative stress. *Neurobiol. Stress* 9 9–21. 10.1016/j.ynstr.2018.05.003 29992181 PMC6035903

[B18] Brenowitz W. D. Besser L. M. Kukull W. A. Keene C. D. Glymour M. M. Yaffe K. (2020). Clinician-judged hearing impairment and associations with neuropathologic burden. *Neurology* 95 e1640–e1649. 10.1212/WNL.0000000000010575 32759190 PMC7713726

[B19] Campeau S. Watson S. J. Jr. (2000). Connections of some auditory-responsive posterior thalamic nuclei putatively involved in activation of the hypothalamo-pituitary-adrenocortical axis in response to audiogenic stress in rats: An anterograde and retrograde tract tracing study combined with Fos expression. *J. Comp. Neurol.* 423 474–491. 10870087

[B20] Canlon B. Meltser I. Johansson P. Tahera Y. (2007). Glucocorticoid receptors modulate auditory sensitivity to acoustic trauma. *Hear. Res.* 226 61–69. 10.1016/j.heares.2006.05.009 16843624

[B21] Canlon B. Theorell T. Hasson D. (2013). Associations between stress and hearing problems in humans. *Hear. Res.* 295 9–15. 10.1016/j.heares.2012.08.015 22982334

[B22] Cardon E. Joossen I. Vermeersch H. Jacquemin L. Mertens G. Vanderveken O. M. (2020). Systematic review and meta-analysis of late auditory evoked potentials as a candidate biomarker in the assessment of tinnitus. *PLoS One* 15:e0243785. 10.1371/journal.pone.0243785 33332414 PMC7746183

[B23] Chattarji S. Tomar A. Suvrathan A. Ghosh S. Rahman M. M. (2015). Neighborhood matters: Divergent patterns of stress-induced plasticity across the brain. *Nat. Neurosci.* 18 1364–1375. 10.1038/nn.4115 26404711

[B24] Chen D. S. Genther D. J. Betz J. Lin F. R. (2014). Association between hearing impairment and self-reported difficulty in physical functioning. *J. Am. Geriatr. Soc.* 62 850–856. 10.1111/jgs.12800 24779559 PMC4084895

[B25] Chen H. Kwong J. C. Copes R. Tu K. Villeneuve P. J. van Donkelaar A. (2017). Living near major roads and the incidence of dementia. Parkinson’s disease, and multiple sclerosis: A population-based cohort study. *Lancet* 389 718–726. 10.1016/S0140-6736(16)32399-6 28063597

[B26] Chen J. J. Zeng B. S. Wu C. N. Stubbs B. Carvalho A. F. Brunoni A. R. (2020). Association of central noninvasive brain stimulation interventions with efficacy and safety in tinnitus management: A meta-analysis. *JAMA Otolaryngol. Head Neck Surg.* 146 801–809. 10.1001/jamaoto.2020.1497 32644131 PMC7349076

[B27] Clegg A. Young J. Iliffe S. Rikkert M. O. Rockwood K. (2013). Frailty in elderly people. *Lancet* 381 752–762. 10.1016/S0140-6736(12)62167-9 23395245 PMC4098658

[B28] Cui B. Zhu L. She X. Wu M. Ma Q. Wang T. (2012). Chronic noise exposure causes persistence of tau hyperphosphorylation and formation of NFT tau in the rat hippocampus and prefrontal cortex. *Exp. Neurol.* 238 122–129. 10.1016/j.expneurol.2012.08.028 22971273

[B29] Cullati S. Kliegel M. Widmer E. (2018). Development of reserves over the life course and onset of vulnerability in later life. *Nat. Hum. Behav.* 2 551–558. 10.1038/s41562-018-0395-3 31209322

[B30] Cullinan W. E. Herman J. P. Watson S. J. (1993). Ventral subicular interaction with the hypothalamic paraventricular nucleus: Evidence for a relay in the bed nucleus of the stria terminalis. *J. Comp. Neurol.* 332 1–20. 10.1002/cne.903320102 7685778

[B31] De Ridder D. Elgoyhen A. B. Romo R. Langguth B. (2011). Phantom percepts: Tinnitus and pain as persisting aversive memory networks. *Proc. Natl Acad. Sci. U.S.A.* 108 8075–8080. 10.1073/pnas.1018466108 21502503 PMC3100980

[B32] Duman R. S. Aghajanian G. K. Sanacora G. Krystal J. H. (2016). Synaptic plasticity and depression: New insights from stress and rapid-acting antidepressants. *Nat. Med.* 22 238–249. 10.1038/nm.4050 26937618 PMC5405628

[B33] Duman R. S. Sanacora G. Krystal J. H. (2019). Altered connectivity in depression: GABA and glutamate neurotransmitter deficits and reversal by novel treatments. *Neuron* 102 75–90. 10.1016/j.neuron.2019.03.013 30946828 PMC6450409

[B34] Engineer N. D. Riley J. R. Seale J. D. Vrana W. A. Shetake J. A. Sudanagunta S. P. (2011). Reversing pathological neural activity using targeted plasticity. *Nature.* 470 101–104. 10.1038/nature09656 21228773 PMC3295231

[B35] Evans W. J. Lambert C. P. (2007). Physiological basis of fatigue. *Am. J. Phys. Med. Rehabil.* 86 (Suppl. 1), S29–S46. 10.1097/phm.0b013e31802ba53c 17370370

[B36] Fetoni A. R. Troiani D. Petrosini L. Paludetti G. (2015). Cochlear injury and adaptive plasticity of the auditory cortex. *Front. Aging Neurosci.* 7:8. 10.3389/fnagi.2015.00008 25698966 PMC4318425

[B37] Fried L. P. Tangen C. M. Walston J. Newman A. B. Hirsch C. Gottdiener J. (2001). Frailty in older adults: Evidence for a phenotype. *J. Gerontol. A Biol. Sci. Med. Sci.* 56 M146–M156. 10.1093/gerona/56.3.m146 11253156

[B38] Froemke R. C. Martins A. R. (2011). Spectrotemporal dynamics of auditory cortical synaptic receptive field plasticity. *Hear. Res.* 279 149–161. 10.1016/j.heares.2011.03.005 21426927 PMC3138852

[B39] Furuta H. Mori N. Sato C. Hoshikawa H. Sakai S. Iwakura S. (1994). Mineralocorticoid type I receptor in the rat cochlea: mRNA identification by polymerase chain reaction (PCR) and in situ hybridization. *Hear. Res.* 78 175–180. 10.1016/0378-5955(94)90023-x 7982810

[B40] Gai Z. Su D. Wang Y. Li W. Cui B. Li K. (2017). Effects of chronic noise on the corticotropin-releasing factor system in the rat hippocampus: Relevance to Alzheimer’s disease-like tau hyperphosphorylation. *Environ. Health Prev. Med.* 22:79. 10.1186/s12199-017-0686-8 29228900 PMC5725896

[B41] Garrido P. (2011). Aging and stress: Past hypotheses, present approaches and perspectives. *Aging Dis.* 2 80–99. 22396868 PMC3295041

[B42] Gates G. A. Mills J. H. (2005). Presbycusis. *Lancet* 366 1111–1120. 10.1016/S0140-6736(05)67423-5 16182900

[B43] Gjerstad J. K. Lightman S. L. Spiga F. (2018). Role of glucocorticoid negative feedback in the regulation of HPA axis pulsatility. *Stress* 21 403–416. 10.1080/10253890.2018.1470238 29764284 PMC6220752

[B44] Goncharova N. D. (2020). The HPA axis under stress and aging: Individual vulnerability is associated with behavioral patterns and exposure time. *BioEssays* 42:e2000007. 10.1002/bies.202000007 32666621

[B45] Gröschel M. Hubert N. Müller S. Ernst A. Basta D. (2014). Age-dependent changes of calcium related activity in the central auditory pathway. *Exp. Gerontol.* 58 235–243. 10.1016/j.exger.2014.08.014 25176163

[B46] Gu J. W. Herrmann B. S. Levine R. A. Melcher J. R. (2012). Brainstem auditory evoked potentials suggest a role for the ventral cochlear nucleus in tinnitus. *J. Assoc. Res. Otolaryngol.* 13 819–833. 10.1007/s10162-012-0344-1 22869301 PMC3505586

[B47] Haider H. F. Hoare D. J. Ribeiro S. F. Ribeiro D. Caria H. Trigueiros N. (2021). Evidence for biological markers of tinnitus: A systematic review. *Prog. Brain Res.* 262 345–398. 10.1016/bs.pbr.2021.01.022 33931188

[B48] Haider H. F. Ribeiro D. Ribeiro S. F. Trigueiros N. Caria H. Borrego L. (2022). Audiological biomarkers of tinnitus in an older Portuguese population. *Front. Aging Neurosci.* 14:933117. 10.3389/fnagi.2022.933117 36092804 PMC9449802

[B49] Haider H. F. Ribeiro S. F. Martins C. Ribeiro D. Trigueiros N. Szczepek A. J. (2020). Tinnitus, hearing loss and inflammatory processes in an older Portuguese population. *Int. J. Audiol.* 59 323–332. 10.1080/14992027.2019.1698775 31829778

[B50] Hasson D. Theorell T. Wallén M. B. Leineweber C. Canlon B. (2011). Stress and prevalence of hearing problems in the Swedish working population. *BMC Public Health* 11:130. 10.1186/1471-2458-11-130 21345187 PMC3056746

[B51] Hébert S. Paiement P. Lupien S. J. (2004). A physiological correlate for the intolerance to both internal and external sounds. *Hear. Res.* 190 1–9. 10.1016/S0378-5955(04)00021-8 15051125

[B52] Heeringa A. N. Stefanescu R. A. Raphael Y. Shore S. E. (2016). Altered vesicular glutamate transporter distributions in the mouse cochlear nucleus following cochlear insult. *Neuroscience* 315 114–124. 10.1016/j.neuroscience.2015.12.009 26705736 PMC4715966

[B53] Heeringa A. N. Wu C. Chung C. West M. Martel D. Liberman L. (2018). Glutamatergic projections to the cochlear nucleus are redistributed in tinnitus. *Neuroscience* 391 91–103. 10.1016/j.neuroscience.2018.09.008 30236972 PMC6191338

[B54] Henry J. A. Roberts L. E. Caspary D. M. Theodoroff S. M. Salvi R. J. (2014). Underlying mechanisms of tinnitus: Review and clinical implications. *J. Am. Acad. Audiol.* 25 5–22. 10.3766/jaaa.25.1.2 24622858 PMC5063499

[B55] Hequembourg S. Liberman M. C. (2001). Spiral ligament pathology: A major aspect of age-related cochlear degeneration in C57BL/6 mice. *J. Assoc. Res. Otolaryngol.* 2 118–129. 10.1007/s101620010075 11550522 PMC3201181

[B56] Herman J. P. Cullinan W. E. Morano M. I. Akil H. Watson S. J. (1995). Contribution of the ventral subiculum to inhibitory regulation of the hypothalamo-pituitary-adrenocortical axis. *J. Neuroendocrinol.* 7 475–482. 10.1111/j.1365-2826.1995.tb00784.x 7550295

[B57] Herman J. P. McKlveen J. M. Ghosal S. Kopp B. Wulsin A. Makinson R. (2016). Regulation of the hypothalamic-pituitary-adrenocortical stress response. *Compr. Physiol.* 6 603–621. 10.1002/cphy.c150015 27065163 PMC4867107

[B58] Hildesheimer M. Sharon R. Muchnik C. Sahartov E. Rubinstein M. (1991). The effect of bilateral sympathectomy on noise induced temporary threshold shift. *Hear. Res.* 51 49–53. 10.1016/0378-5955(91)90006-u 2013545

[B59] Hoogendijk E. O. Afilalo J. Ensrud K. E. Kowal P. Onder G. Fried L. P. (2019). Frailty: Implications for clinical practice and public health. *Lancet* 394 1365–1375. 10.1016/S0140-6736(19)31786-6 31609228

[B60] Ibrahim B. A. Llano D. A. (2019). Aging and central auditory disinhibition: Is it a reflection of homeostatic downregulation or metabolic vulnerability? *Brain Sci.* 9:351. 10.3390/brainsci9120351 31805729 PMC6955996

[B61] Jackson J. G. (2019). The cortisol awakening response: A feasibility study investigating the use of the area under the curve with respect to increase as an effective objective measure of tinnitus distress. *Am. J. Audiol.* 28 583–596. 10.1044/2019_AJA-18-0174 31318575

[B62] Jafari Z. Kolb B. E. Mohajerani M. H. (2020b). Noise exposure accelerates the risk of cognitive impairment and Alzheimer’s disease: Adulthood, gestational, and prenatal mechanistic evidence from animal studies. *Neurosci. Biobehav. Rev.* 117 110–128. 10.1016/j.neubiorev.2019.04.001 30978359

[B63] Jafari Z. Copps T. Hole G. Kolb B. E. Mohajerani M. H. (2020a). Noise damage accelerates auditory aging and tinnitus: A Canadian population-based study. *Otol. Neurotol.* 41 1316–1326. 10.1097/MAO.0000000000002848 32810017

[B64] Jafari Z. Copps T. Hole G. Nyatepe-Coo F. Kolb B. E. Mohajerani M. H. (2022). Tinnitus, sound intolerance, and mental health: The role of long-term occupational noise exposure. *Eur. Arch. Otorhinolaryngol.* 279 5161–5170. 10.1007/s00405-022-07362-2 35359185

[B65] Jafari Z. Kolb B. E. Mohajerani M. H. (2019). Age-related hearing loss and tinnitus, dementia risk, and auditory amplification outcomes. *Ageing Res. Rev.* 56:100963. 10.1016/j.arr.2019.100963 31557539

[B66] Jastreboff P. J. Hazell J. W. (1993). A neurophysiological approach to tinnitus: Clinical implications. *Br. J. Audiol.* 27 7–17. 10.3109/03005369309077884 8339063

[B67] Joo Y. H. Han K. D. Park K. H. (2015). Association of hearing loss and tinnitus with health-related quality of life: The Korea national health and nutrition examination survey. *PLoS One* 10:e0131247. 10.1371/journal.pone.0131247 26121026 PMC4488242

[B68] Kalappa B. I. Brozoski T. J. Turner J. G. Caspary D. M. (2014). Single unit hyperactivity and bursting in the auditory thalamus of awake rats directly correlates with behavioural evidence of tinnitus. *J. Physiol.* 592 5065–5078. 10.1113/jphysiol.2014.278572 25217380 PMC4259543

[B69] Kamil R. J. Betz J. Powers B. B. Pratt S. Kritchevsky S. Ayonayon H. N. (2016). Association of hearing impairment with incident frailty and falls in older adults. *J. Aging Health* 28 644–660. 10.1177/0898264315608730 26438083 PMC5644033

[B70] Karatsoreos I. N. Bhagat S. M. Bowles N. P. Weil Z. M. Pfaff D. W. McEwen B. S. (2010). Endocrine and physiological changes in response to chronic corticosterone: A potential model of the metabolic syndrome in mouse. *Endocrinology* 151 2117–2127. 10.1210/en.2009-1436 20211972 PMC2869265

[B71] Kim Y. K. Na K. S. Myint A. M. Leonard B. E. (2016). The role of pro-inflammatory cytokines in neuroinflammation, neurogenesis and the neuroendocrine system in major depression. *Prog. Neuropsychopharmacol. Biol. Psychiatry* 64 277–284. 10.1016/j.pnpbp.2015.06.008 26111720

[B72] Knipper M. Van Dijk P. Nunes I. Rüttiger L. Zimmermann U. (2013). Advances in the neurobiology of hearing disorders: Recent developments regarding the basis of tinnitus and hyperacusis. *Prog. Neurobiol.* 111 17–33. 10.1016/j.pneurobio.2013.08.002 24012803

[B73] Koehler S. D. Shore S. E. (2013). Stimulus timing-dependent plasticity in dorsal cochlear nucleus is altered in tinnitus. *J. Neurosci.* 33 19647–19656. 10.1523/JNEUROSCI.2788-13.2013 24336728 PMC3858633

[B74] Kraus K. S. Canlon B. (2012). Neuronal connectivity and interactions between the auditory and limbic systems. Effects of noise and tinnitus. *Hear. Res.* 288 34–46. 10.1016/j.heares.2012.02.009 22440225

[B75] Krizman J. Skoe E. Kraus N. (2016). Bilingual enhancements have no socioeconomic boundaries. *Dev. Sci.* 19 881–891. 10.1111/desc.12347 26573107

[B76] Kroll J. F. Dussias P. E. Bice K. Perrotti L. (2015). Bilingualism, mind, and brain. *Annu. Rev. Linguist.* 1 377–394. 10.1146/annurev-linguist-030514-124937 28642932 PMC5478196

[B77] Kuh D. Karunananthan S. Bergman H. Cooper R. (2014). A life-course approach to healthy ageing: Maintaining physical capability. *Proc. Nutr. Soc.* 73 237–248. 10.1017/S0029665113003923 24456831 PMC3981474

[B78] Kujawa S. G. Liberman M. C. (2009). Adding insult to injury: Cochlear nerve degeneration after “temporary” noise-induced hearing loss. *J. Neurosci.* 29 14077–14085. 10.1523/JNEUROSCI.2845-09.2009 19906956 PMC2812055

[B79] Kujawa S. G. Liberman M. C. (2015). Synaptopathy in the noise-exposed and aging cochlea: Primary neural degeneration in acquired sensorineural hearing loss. *Hear. Res.* 330 191–199. 10.1016/j.heares.2015.02.009 25769437 PMC4567542

[B80] Langguth B. Landgrebe M. Kleinjung T. Sand G. P. Hajak G. (2011). Tinnitus and depression. *World J. Biol. Psychiatry* 12 489–500. 10.3109/15622975.2011.575178 21568629

[B81] Langguth B. Schecklmann M. Lehner A. Landgrebe M. Poeppl T. B. Kreuzer P. M. (2012). Neuroimaging and neuromodulation: Complementary approaches for identifying the neuronal correlates of tinnitus. *Front. Syst. Neurosci.* 6:15. 10.3389/fnsys.2012.00015 22509155 PMC3321434

[B82] Lau T. Bigio B. Zelli D. McEwen B. S. Nasca C. (2017). Stress-induced structural plasticity of medial amygdala stellate neurons and rapid prevention by a candidate antidepressant. *Mol. Psychiatry* 22 227–234. 10.1038/mp.2016.68 27240534 PMC5133196

[B83] Lee C. H. Giuliani F. (2019). The role of inflammation in depression and fatigue. *Front. Immunol.* 10:1696. 10.3389/fimmu.2019.01696 31379879 PMC6658985

[B84] Li S. Kalappa B. I. Tzounopoulos T. (2015). Noise-induced plasticity of KCNQ2/3 and HCN channels underlies vulnerability and resilience to tinnitus. *eLife* 4:e07242. 10.7554/eLife.07242 26312501 PMC4592936

[B85] Liberman M. C. (1978). Auditory-nerve response from cats raised in a low-noise chamber. *J. Acoust. Soc. Am.* 63 442–455. 10.1121/1.381736 670542

[B86] Liberman M. C. Kujawa S. G. (2017). Cochlear synaptopathy in acquired sensorineural hearing loss: Manifestations and mechanisms. *Hear. Res.* 349 138–147. 10.1016/j.heares.2017.01.003 28087419 PMC5438769

[B87] Loughrey D. G. Feeney J. Kee F. Lawlor B. A. Woodside J. V. Setti A. (2021). Social factors may mediate the relationship between subjective age-related hearing loss and episodic memory. *Aging Ment. Health* 25 824–831. 10.1080/13607863.2020.1727847 32067488

[B88] Lozupone M. Sardone R. Panza F. (2020). Age-related hearing loss and neuropathologic burden: A step inside the cognitive ear. *Neurology* 95 511–512. 10.1212/WNL.0000000000010580 32759199

[B89] Maharani A. Pendleton N. Leroi I. (2019). Hearing impairment, loneliness, social isolation, and cognitive function: Longitudinal analysis using English longitudinal study on ageing. *Am. J. Geriatr. Psychiatry* 27 1348–1356. 10.1016/j.jagp.2019.07.010 31402088

[B90] Manohar S. Chen G. D. Ding D. Liu L. Wang J. Chen Y. C. (2022). Unexpected consequences of noise-induced hearing loss: Impaired hippocampal neurogenesis, memory, and stress. *Front. Integr. Neurosci.* 16:871223. 10.3389/fnint.2022.871223 35619926 PMC9127992

[B91] Marks K. L. Martel D. T. Wu C. Basura G. J. Roberts L. E. Schvartz-Leyzac K. C. (2018). Auditory-somatosensory bimodal stimulation desynchronizes brain circuitry to reduce tinnitus in guinea pigs and humans. *Sci. Transl. Med.* 10:eaal3175. 10.1126/scitranslmed.aal3175 29298868 PMC5863907

[B92] Marsh R. A. Fuzessery Z. M. Grose C. D. Wenstrup J. J. (2002). Projection to the inferior colliculus from the basal nucleus of the amygdala. *J. Neurosci.* 22 10449–10460. 10.1523/JNEUROSCI.22-23-10449.2002 12451144 PMC6758740

[B93] Marsland A. L. Gianaros P. J. Abramowitch S. M. Manuck S. B. Hariri A. R. (2008). Interleukin-6 covaries inversely with hippocampal grey matter volume in middle-aged adults. *Biol. Psychiatry* 64 484–490. 10.1016/j.biopsych.2008.04.016 18514163 PMC2562462

[B94] Mazurek B. Haupt H. Olze H. Szczepek A. J. (2012). Stress and tinnitus-from bedside to bench and back. *Front. Syst. Neurosci.* 6:47. 10.3389/fnsys.2012.00047 22701404 PMC3371598

[B95] McEwen B. S. (1998). Stress, adaptation, and disease. Allostasis and allostatic load. *Ann. N. Y. Acad. Sci.* 840 33–44. 10.1111/j.1749-6632.1998.tb09546.x 9629234

[B96] McEwen B. S. Akil H. (2020). Revisiting the stress concept: Implications for affective disorders. *J. Neurosci.* 40 12–21. 10.1523/JNEUROSCI.0733-19.2019 31896560 PMC6939488

[B97] McEwen B. S. Nasca C. Gray J. D. (2016). Stress effects on neuronal structure: Hippocampus, amygdala, and prefrontal cortex. *Neuropsychopharmacology* 41 3–23. 10.1038/npp.2015.171 26076834 PMC4677120

[B98] Middleton J. W. Kiritani T. Pedersen C. Turner J. G. Shepherd G. M. Tzounopoulos T. (2011). Mice with behavioral evidence of tinnitus exhibit dorsal cochlear nucleus hyperactivity because of decreased GABAergic inhibition. *Proc. Natl Acad. Sci. U.S.A.* 108 7601–7606. 10.1073/pnas.1100223108 21502491 PMC3088638

[B99] Miller A. L. Lumeng J. C. (2018). Pathways of association from stress to obesity in early childhood. *Obesity* 26 1117–1124. 10.1002/oby.22155 29656595

[B100] Oberlander T. F. Weinberg J. Papsdorf M. Grunau R. Misri S. Devlin A. M. (2008). Prenatal exposure to maternal depression, neonatal methylation of human glucocorticoid receptor gene (NR3C1) and infant cortisol stress responses. *Epigenetics.* 3 97–106. 10.4161/epi.3.2.6034 18536531

[B101] Panza F. Solfrizzi V. Seripa D. Imbimbo B. P. Capozzo R. Quaranta N. (2015). Age-related hearing impairment and frailty in Alzheimer’s disease: Interconnected associations and mechanisms. *Front. Aging Neurosci.* 7:113. 10.3389/fnagi.2015.00113 26106327 PMC4460423

[B102] Patel D. Anilkumar S. Chattarji S. Buwalda B. (2018). Repeated social stress leads to contrasting patterns of structural plasticity in the amygdala and hippocampus. *Behav. Brain Res.* 347 314–324. 10.1016/j.bbr.2018.03.034 29580891

[B103] Pattyn T. Van Den Eede F. Vanneste S. Cassiers L. Veltman D. J. Van De Heyning P. (2016). Tinnitus and anxiety disorders: A review. *Hear. Res.* 333 255–265. 10.1016/j.heares.2015.08.014 26342399

[B104] Patuzzi R. (2002). Non-linear aspects of outer hair cell transduction and the temporary threshold shifts after acoustic trauma. *Audiol. Neurootol.* 7 17–20. 10.1159/000046857 11914520

[B105] Phillips D. I. Barker D. J. Fall C. H. Seckl J. R. Whorwood C. B. Wood P. J. (1998). Elevated plasma cortisol concentrations: A link between low birth weight and the insulin resistance syndrome? *J. Clin. Endocrinol. Metab.* 83 757–760. 10.1210/jcem.83.3.4634 9506721

[B106] Picard M. McEwen B. S. Epel E. S. Sandi C. (2018). An energetic view of stress: Focus on mitochondria. *Front. Neuroendocrinol.* 49 72–85. 10.1016/j.yfrne.2018.01.001 29339091 PMC5964020

[B107] Piccirillo J. F. Rodebaugh T. L. Lenze E. J. (2020). Tinnitus. *JAMA.* 323 1497–1498. 10.1001/jama.2020.0697 32176246

[B108] Popoli M. Yan Z. McEwen B. S. Sanacora G. (2011). The stressed synapse: The impact of stress and glucocorticoids on glutamate transmission. *Nat. Rev. Neurosci.* 13 22–37. 10.1038/nrn3138 22127301 PMC3645314

[B109] Price R. B. Duman R. (2020). Neuroplasticity in cognitive and psychological mechanisms of depression: An integrative model. *Mol. Psychiatry* 25 530–543. 10.1038/s41380-019-0615-x 31801966 PMC7047599

[B110] Quirarte G. L. Roozendaal B. McGaugh J. L. (1997). Glucocorticoid enhancement of memory storage involves noradrenergic activation in the basolateral amygdala. *Proc. Natl. Acad. Sci. U.S.A.* 94 14048–14053. 10.1073/pnas.94.25.14048 9391150 PMC28430

[B111] Rao R. Androulakis I. P. (2019). The physiological significance of the circadian dynamics of the HPA axis: Interplay between circadian rhythms, allostasis and stress resilience. *Horm. Behav.* 110 77–89. 10.1016/j.yhbeh.2019.02.018 30862458

[B112] Reichardt L. F. (2006). Neurotrophin-regulated signalling pathways. *Philos. Trans. R. Soc. Lond. B Biol. Sci.* 361 1545–1564. 10.1098/rstb.2006.1894 16939974 PMC1664664

[B113] Roesler R. Parent M. B. LaLumiere R. T. McIntyre C. K. (2021). Amygdala-hippocampal interactions in synaptic plasticity and memory formation. *Neurobiol. Learn. Mem.* 184:107490. 10.1016/j.nlm.2021.107490 34302951 PMC8435011

[B114] Roozendaal B. McEwen B. S. Chattarji S. (2009). Stress, memory and the amygdala. *Nat. Rev. Neurosci.* 10 423–433. 10.1038/nrn2651 19469026

[B115] Ruan Q. Chen J. Zhang R. Zhang W. Ruan J. Zhang M. (2021). Heterogeneous influence of frailty phenotypes in age-related hearing loss and tinnitus in Chinese older adults: An explorative study. *Front. Psychol.* 11:617610. 10.3389/fpsyg.2020.617610 33664689 PMC7921692

[B116] Ruan Q. Yu Z. Chen M. Bao Z. Li J. He W. (2015). Cognitive frailty, a novel target for the prevention of elderly dependency. *Ageing Res. Rev.* 20 1–10. 10.1016/j.arr.2014.12.004 25555677

[B117] Rüttiger L. Singer W. Panford-Walsh R. Matsumoto M. Lee S. C. Zuccotti A. (2013). The reduced cochlear output and the failure to adapt the central auditory response causes tinnitus in noise exposed rats. *PLoS One* 8:e57247. 10.1371/journal.pone.0057247 23516401 PMC3596376

[B118] Sametsky E. A. Turner J. G. Larsen D. Ling L. Caspary D. M. (2015). Enhanced GABAA-mediated tonic inhibition in auditory thalamus of rats with behavioral evidence of tinnitus. *J. Neurosci.* 35 9369–9380. 10.1523/JNEUROSCI.5054-14.2015 26109660 PMC4478253

[B119] Schaette R. McAlpine D. (2011). Tinnitus with a normal audiogram: Physiological evidence for hidden hearing loss and computational model. *J. Neurosci.* 31 13452–13457. 10.1523/JNEUROSCI.2156-11.2011 21940438 PMC6623281

[B120] Schwabe L. Joëls M. Roozendaal B. Wolf O. T. Oitzl M. S. (2012). Stress effects on memory: An update and integration. *Neurosci. Biobehav. Rev.* 36 1740–1749. 10.1016/j.neubiorev.2011.07.002 21771612

[B121] Seicol B. J. Lin S. Xie R. (2022). Age-related hearing loss is accompanied by chronic inflammation in the cochlea and the cochlear nucleus. *Front. Aging Neurosci.* 14:846804. 10.3389/fnagi.2022.846804 35418849 PMC8995794

[B122] Shen Y. Hu H. Fan C. Wang Q. Zou T. Ye B. (2021). Sensorineural hearing loss may lead to dementia-related pathological changes in hippocampal neurons. *Neurobiol. Dis.* 156:105408. 10.1016/j.nbd.2021.105408 34082124

[B123] Shonkoff J. P. Boyce W. T. McEwen B. S. (2009). Neuroscience, molecular biology, and the childhood roots of health disparities: Building a new framework for health promotion and disease prevention. *JAMA* 301 2252–2259. 10.1001/jama.2009.754 19491187

[B124] Shore S. Zhou J. Koehler S. (2007). Neural mechanisms underlying somatic tinnitus. *Prog. Brain Res.* 166 107–123. 10.1016/S0079-6123(07)66010-5 17956776 PMC2566901

[B125] Shore S. E. Roberts L. E. Langguth B. (2016). Maladaptive plasticity in tinnitus–triggers, mechanisms and treatment. *Nat. Rev. Neurol.* 12 150–160. 10.1038/nrneurol.2016.12 26868680 PMC4895692

[B126] Simoes J. P. Daoud E. Shabbir M. Amanat S. Assouly K. Biswas R. (2021). Multidisciplinary tinnitus research: Challenges and future directions from the perspective of early stage researchers. *Front. Aging Neurosci.* 13:647285. 10.3389/fnagi.2021.647285 34177549 PMC8225955

[B127] Singer W. Zuccotti A. Jaumann M. Lee S. C. Panford-Walsh R. Xiong H. (2013). Noise-induced inner hair cell ribbon loss disturbs central arc mobilization: A novel molecular paradigm for understanding tinnitus. *Mol. Neurobiol.* 47 261–279. 10.1007/s12035-012-8372-8 23154938

[B128] Skoe E. Kraus N. (2014). Auditory reserve and the legacy of auditory experience. *Brain Sci.* 4 575–593. 10.3390/brainsci4040575 25405381 PMC4279143

[B129] Solas M. Aisa B. Mugueta M. C. Del Río J. Tordera R. M. Ramírez M. J. (2010). Interactions between age, stress and insulin on cognition: Implications for Alzheimer’s disease. *Neuropsychopharmacology* 35 1664–1673. 10.1038/npp.2010.13 20182419 PMC3055481

[B130] Solfrizzi V. Scafato E. Lozupone M. Seripa D. Schilardi A. Custodero C. (2019). Biopsychosocial frailty and the risk of incident dementia: The Italian longitudinal study on aging. *Alzheimers Dement.* 15 1019–1028. 10.1016/j.jalz.2019.04.013 31278052

[B131] Stern Y. Barnes C. A. Grady C. Jones R. N. Raz N. (2019). Brain reserve, cognitive reserve, compensation, and maintenance: Operationalization, validity, and mechanisms of cognitive resilience. *Neurobiol. Aging* 83 124–129. 10.1016/j.neurobiolaging.2019.03.022 31732015 PMC6859943

[B132] Szczepek A. J. Stankovic K. M. (2021). Editorial: Emerging ototoxic medications and their role in cochlear and vestibular disorders. *Front. Neurol.* 12:773714. 10.3389/fneur.2021.773714 34744994 PMC8569918

[B133] Tai X. Y. Veldsman M. Lyall D. M. Littlejohns T. J. Langa K. M. Husain M. (2022). Cardiometabolic multimorbidity, genetic risk, and dementia: A prospective cohort study. *Lancet Healthy Longev.* 3 e428–e436. 10.1016/S2666-7568(22)00117-9 35711612 PMC9184258

[B134] Tatomir A. Micu C. Crivii C. (2014). The impact of stress and glucocorticoids on memory. *Clujul Med.* 87 3–6. 10.15386/cjm.2014.8872.871.at1cm2 26527987 PMC4462413

[B135] Thomas W. Harvey B. J. (2011). Mechanisms underlying rapid aldosterone effects in the kidney. *Annu. Rev. Physiol.* 73 335–357. 10.1146/annurev-physiol-012110-142222 20809792

[B136] Tian R. Trevenen M. Ford A. H. Jayakody D. M. P. Hankey G. J. Yeap B. B. (2022). Hearing impairment and frailty in later life: The Health in Men Study (HIMS). *Maturitas* 156 30–36. 10.1016/j.maturitas.2021.10.008 35033231

[B137] Valenzuela M. J. Sachdev P. (2006). Brain reserve and dementia: A systematic review. *Psychol. Med.* 36 441–454. 10.1017/S0033291705006264 16207391

[B138] van Gendt M. J. Boyen K. de Kleine E. Langers D. R. van Dijk P. (2012). The relation between perception and brain activity in gaze-evoked tinnitus. *J. Neurosci.* 32 17528–17539. 10.1523/JNEUROSCI.2791-12.2012 23223277 PMC6621667

[B139] Vyas A. Mitra R. Shankaranarayana Rao B. S. Chattarji S. (2002). Chronic stress induces contrasting patterns of dendritic remodeling in hippocampal and amygdaloid neurons. *J. Neurosci.* 22 6810–6818. 12151561 10.1523/JNEUROSCI.22-15-06810.2002 PMC6758130

[B140] Wan G. Corfas G. (2017). Transient auditory nerve demyelination as a new mechanism for hidden hearing loss. *Nat. Commun.* 8:14487. 10.1038/ncomms14487 28211470 PMC5321746

[B141] Wang H. Brozoski T. J. Caspary D. M. (2011). Inhibitory neurotransmission in animal models of tinnitus: Maladaptive plasticity. *Hear. Res.* 279 111–117. 10.1016/j.heares.2011.04.004 21527325 PMC3172385

[B142] Waters R. L. Lunsford B. R. Perry J. Byrd R. (1988). Energy-speed relationship of walking: Standard tables. *J. Orthop. Res.* 6 215–222. 10.1002/jor.1100060208 3343627

[B143] Watson N. Ding B. Zhu X. Frisina R. D. (2017). Chronic inflammation - inflammaging - in the ageing cochlea: A novel target for future presbycusis therapy. *Ageing Res. Rev.* 40 142–148. 10.1016/j.arr.2017.10.002 29017893 PMC5675822

[B144] Wayne R. V. Johnsrude I. S. (2015). A review of causal mechanisms underlying the link between age-related hearing loss and cognitive decline. *Ageing Res. Rev.* 23 154–166. 10.1016/j.arr.2015.06.002 26123097

[B145] Weaver I. C. Cervoni N. Champagne F. A. D’Alessio A. C. Sharma S. Seckl J. R. (2004). Epigenetic programming by maternal behavior. *Nat. Neurosci.* 7 847–854. 10.1038/nn1276 15220929

[B146] Weikum E. R. Knuesel M. T. Ortlund E. A. Yamamoto K. R. (2017). Glucocorticoid receptor control of transcription: Precision and plasticity via allostery. *Nat. Rev. Mol. Cell Biol.* 18 159–174. 10.1038/nrm.2016.152 28053348 PMC6257982

[B147] Wilkinson C. W. Petrie E. C. Murray S. R. Colasurdo E. A. Raskind M. A. Peskind E. R. (2001). Human glucocorticoid feedback inhibition is reduced in older individuals: Evening study. *J. Clin. Endocrinol. Metab.* 86 545–550. 10.1210/jcem.86.2.7232 11158007

[B148] Wu C. Martel D. T. Shore S. E. (2016). Increased synchrony and bursting of dorsal cochlear nucleus fusiform cells correlate with tinnitus. *J. Neurosci.* 36 2068–2073. 10.1523/JNEUROSCI.3960-15.2016 26865628 PMC4748084

[B149] Xu W. Zhang C. Li J. Q. Tan C. C. Cao X. P. Tan L. (2019). Age-related hearing loss accelerates cerebrospinal fluid tau levels and brain atrophy: A longitudinal study. *Aging* 11 3156–3169. 10.18632/aging.101971 31118310 PMC6555452

[B150] Yamada M. Arai H. (2018). Social frailty predicts incident disability and mortality among community-dwelling Japanese older adults. *J. Am. Med. Dir. Assoc.* 19 1099–1103. 10.1016/j.jamda.2018.09.013 30471801

[B151] Yau P. L. Castro M. G. Tagani A. Tsui W. H. Convit A. (2012). Obesity and metabolic syndrome and functional and structural brain impairments in adolescence. *Pediatrics* 130 e856–e864. 10.1542/peds.2012-0324 22945407 PMC3457620

[B152] Yévenes-Briones H. Caballero F. F. Struijk E. A. Rey-Martinez J. Montes-Jovellar L. Graciani A. (2021). Association between hearing loss and impaired physical function, frailty, and disability in older adults: A cross-sectional study. *JAMA Otolaryngol. Head Neck Surg.* 147 951–958. 10.1001/jamaoto.2021.2399 34554203 PMC8461549

[B153] Yoo M. Kim S. Kim B. S. Yoo J. Lee S. Jang H. C. (2019). Moderate hearing loss is related with social frailty in a community-dwelling older adults: The Korean Frailty and Aging Cohort Study (KFACS). *Arch. Gerontol. Geriatr.* 83 126–130. 10.1016/j.archger.2019.04.004 31003135

[B154] Yu Y. F. Zhai F. Dai C. F. Hu J. J. (2011). The relationship between age-related hearing loss and synaptic changes in the hippocampus of C57BL/6J mice. *Exp. Gerontol.* 46 716–722. 10.1016/j.exger.2011.04.007 21586320

[B155] Zahodne L. B. Stern Y. Manly J. J. (2015). Differing effects of education on cognitive decline in diverse elders with low versus high educational attainment. *Neuropsychology* 29 649–657. 10.1037/neu0000141 25222199 PMC4362867

[B156] Zhang L. Wu C. Martel D. T. West M. Sutton M. A. Shore S. E. (2019). Remodeling of cholinergic input to the hippocampus after noise exposure and tinnitus induction in Guinea pigs. *Hippocampus* 29 669–682. 10.1002/hipo.23058 30471164 PMC7357289

[B157] Zhang L. Wu C. Martel D. T. West M. Sutton M. A. Shore S. E. (2021). Noise exposure alters glutamatergic and GABAergic synaptic connectivity in the hippocampus and its relevance to tinnitus. *Neural Plast.* 2021:8833087. 10.1155/2021/8833087 33510780 PMC7822664

[B158] Zhang W. Ruan J. Zhang R. Zhang M. Hu X. Yu Z. (2021). Age-related hearing loss with tinnitus and physical frailty influence the overall and domain-specific quality of life of Chinese community-dwelling older adults. *Front. Med.* 8:762556. 10.3389/fmed.2021.762556 34746196 PMC8567022

[B159] Zhong S. X. Liu Z. H. (2009). Expression patterns of Nedd4 isoforms and SGK1 in the rat cochlea. *Acta Otolaryngol.* 129 935–939. 10.1080/00016480802552501 19051070

